# Critical Partnerships: How to Develop a Trans-Disciplinary Research Team

**DOI:** 10.3390/cancers15205078

**Published:** 2023-10-20

**Authors:** Kristin A. Waite, Peter J. Pronovost, Jill S. Barnholtz-Sloan

**Affiliations:** 1 Trans Divisional Research Program, Division of Cancer Epidemiology and Genetics, National Cancer Institute, Bethesda, MD 20892, USA; 2 University Hospitals Health System, Shaker Heights, OH 44106, USA; 3 UH Quality Care Organization and UH Accountable Care Organization, University Hospitals Health System, Shaker Heights, OH 44106, USA; 4 Center for Biomedical Informatics and Information Technology, National Cancer Institute, Bethesda, MD 20892, USA

**Keywords:** team science, trans-disciplinary teams, clinical partnership

## Abstract

**Simple Summary:**

Trans-disciplinary team science is critical for advancing our understanding and treatment of cancer. A successful scientific team requires working with a diverse group of individuals from basic researchers to clinicians, bioinformaticians, and many others. Each team member has a different expertise and access and is brought together to work toward a shared goal. While the importance of team science has become evident, these teams do not come together easily or randomly. Here, we aim to provide a concise, high-level view by providing some practical tips to help cancer researchers be successful in a trans-disciplinary team.

**Abstract:**

Trans-disciplinary science will continue to be critical for the next wave of scientific advancement to fully understand cancer development, progression, and treatment. The shift from the independent investigator to either leading or being a productive member of a scientific team can be successful by focusing on some key elements that can build and strengthen interactions with a diverse group of people. These include the selection of the team, communication, leadership and mentorship, shared goals, responsibility to the team, authorship, and proactively dealing with conflict. While there are extensive books written on developing teams in the business world, and larger pieces in the medical arena, we attempt to provide here a concise, high-level view as a starting point for those that may be moving from being an independent researcher and are developing their own, larger, trans-disciplinary teams.

## 1. The Movement toward Trans-Disciplinary Team Science

The image of a scientist has stereotypically been the single Principal Investigator (PI) and their individual lab. Each PI and their lab work in a silo churning out papers, data, and ideas to drive science incrementally forward. Yet, most great discoveries emerge not from within a single discipline but from the intersection of disciplines. Donald Stokes highlighted this in Pasteur’s Quadrant, the idea of elucidating knowledge to have an immediate societal impact [1]. This stereotype of the embattled PI with a team of toiling graduate students and post-doctoral fellows began to shift about a decade ago. By working across disciplines, or trans-discipline, innovation is promoted by bringing together diverse teams to work in basic and applied research aiming to develop solutions. This approach can be seen in cancer, moving the organizational research unit from the single lab/investigator to a trans-disciplinary team comprising basic cancer researchers, clinical oncologists, bioinformaticians, and many others, each with different expertise and access, brought together intentionally to work toward a shared goal to impact cancer. 

The shift to an investigator playing a critical role in a trans-disciplinary team, whereby that team would not be successful without their expertise, provides a different type of avenue for an individual investigator to have a scientific impact. Universities began to recognize that working across disciplines would be critical to the next wave of cutting-edge scientific advancement. In turn, Promotion and Tenure (P&T) standards began to shift. Universities and healthcare systems modified their faculty bylaws and guidance about P&T to recognize “team science” as an acceptable criterion for promotion and tenure. Perhaps more importantly, granting agencies began to develop and shift funding mechanisms from the individual researcher to those favoring a team of researchers. In the United States, the development of collaborative team science funding mechanisms from the National Institutes of Health (NIH) and the National Cancer Institute (NCI), such as multi-PI R01s, Specialized Programs of Research Excellence (SPORE), and the development and funding of NCI-designated Cancer Centers, encouraged the need for investigators to develop into teams. In order for a Cancer Center to gain NCI-designation as a Comprehensive Cancer Center, it must show strength in basic, translational, and clinical research, thereby providing the perfect arena for fostering trans-disciplinary team science.

## 2. The Importance of Trans-Disciplinary Team Science in Cancer

The growing importance of trans-disciplinary team science, both from an intellectual as well as funding viewpoint, has resulted in a significant shift in how science is carried out. Investigators recognize that working across disciplines is critical to the next wave of cutting-edge scientific advancement. The larger team comprises many individuals, from differing areas of research and training, intentionally brought together to address a common hypothesis/goal/endpoint. These goals can be broad and long-term such as elucidating the molecular mechanisms of a disease to develop new treatments. Recent examples of this type of team approach in cancer come from recent and past awardees recognized by the American Association for Cancer Research (AACR). AACR Team Science Awards [2] highlight how teams of clinicians, basic researchers, and varying disciplines can lead to innovations that translate directly to patient care. The 2021 awardees have multiple discoveries in pediatric cancer [2]. This trans-disciplinary team has over 300 manuscripts, 44 patent applications, and clinical trials impacting thousands of pediatric patients [3,4], a feat that would have been difficult for one individual to accomplish. Goals can also be very specific and time-sensitive such as developing a vaccine for a new virus [5] and responding to a global pandemic in the cancer field [6]. These examples of both long- and short-term goals brought together diverse, trans-disciplinary groups of researchers and clinicians, enabling substantial changes in our understanding of disease and the ability to treat patients at a faster pace than if each worked alone. 

While the importance of team science is evident, these teams do not come together easily or randomly. A successful team takes time to come together and function at a high level, and the return on investment can, at first, appear slow during the building stage. In a successful trans-disciplinary team, each member plays a critical role, and their absence diminishes the team’s ability to be successful. Investigators may struggle in developing and effectively managing a trans-disciplinary team. One can look toward the business community to find many approaches to developing and managing a team [7,8,9,10]; however, there are unique aspects to research that may not be covered in such literature. The NCI has written and published an extensive field guide for Collaboration Team Science, which we would direct readers to for an in-depth discussion [11]. 

**Figure 1:**
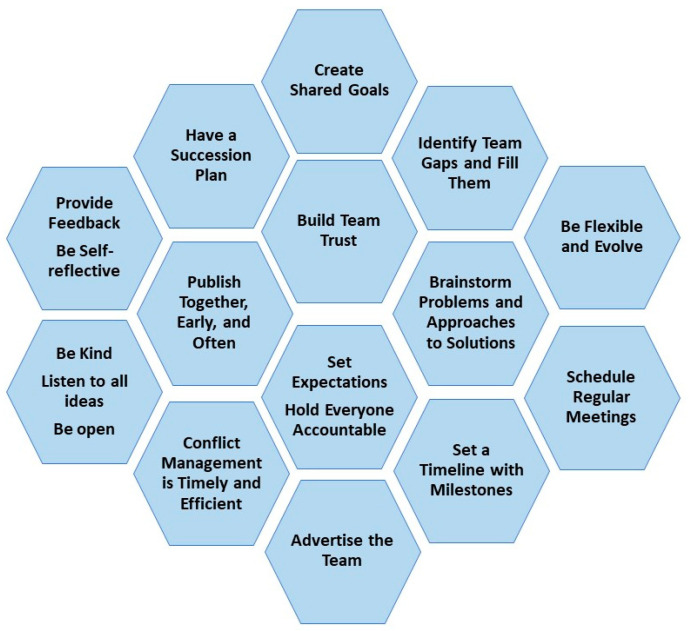
Practical components/approaches utilized in creating and sustaining a successful trans-disciplinary team.

Here, we aim to provide a concise, high-level view by providing some practical tips to help cancer researchers be successful using a trans-disciplinary team science approach (Figure 1). Over years of trial and error, we have found that the key steps to assembling teams, while not complicated, take trust, time, patience, flexibility, and continuous work. The key steps are (1) selecting people to participate, (2) shared goals, (3) accountability, (4) credit, (5) communication, (6) leadership and mentoring, (7) dealing with conflict, and (8) continuing to advance, and we provide a brief overview of each below. In addition, we include a table outlining some common potential challenges with approaches/solutions to address these (Table 1). 

### 2.1. Select People to Participate 

Research teams are unique, varying by organization, location, and size. They may focus on goals beyond scientific breakthroughs including teaching, mentorship, public health policy, and clinical translation [12]. An example of a cancer-focused team initiative that looks beyond scientific discovery is the Persistent Poverty Initiative and the NCI Telehealth Research Centers of Excellence (TRACE), which brings together team members outside of what many consider traditional science [13,14]. The Persistent Poverty Initiative is awarded to five new Centers for Cancer Control in Persistent Poverty Areas to investigate the “structural and institutional factors of persistent poverty in the context of cancer” [15]. Initiatives funded through TRACE will pull together teams with diverse interests and expertise, which may not have ever come together otherwise, to advance our care and treatment of cancer patients. 

A large stumbling block for many basic cancer researchers is gaining access to human biological samples with comprehensive clinical data. Conversely, for the clinical oncologist, a barrier is often the ability to perform bench research experiments. However, by working together, these investigators can advance research both further and faster than working alone. Finding each other may require each to go beyond their initial comfort zone to interact with those outside their discipline. By carefully considering methodological and clinical disciplines that may inform the research effort as well as provide necessary resources, connections to potential collaborators can be identified and developed. 

To develop your own team, we encourage you to be as broad as possible. Think about including social scientists, engineers, diverse health care workers, as well as clinical and basic science researchers. Different backgrounds, languages, and personalities can enhance the team. Members of your team should want to make change and develop meaningful relationships while working toward common goals. Talk with each participant to ensure that they want to engage in team science—they should be humble and curious enough to appreciate the contributions of other disciplines and be eager to actively engage. The most effective team will consist of members and leaders that contribute to the success of the team with a collaborative spirit and willingness to share ideas, data, decision making, and recognition.

### 2.2. Shared Goals

Perhaps the cornerstone of any team, particularly a diverse trans-disciplinary team, is shared goals [16]. As such, these goals should be mutually agreed upon and often revisited to ensure that all team members are continuing their efforts toward the common goal. Goals should be both short- as well as long-term and members should be able to articulate how smaller projects fit into the big picture. By creating and generating a multi-year plan with short-, medium-, and long-term goals and timelines, team leadership will ensure that members are able to maintain focus on the shared team goals. When developing these goals, particularly in the beginning stages of the team, some may be initially uneasy and may feel threatened. This is normal and not unexpected, as most investigators were trained to work independently; therefore, building trust through the shared goals is important for all team members. 

### 2.3. Accountability

A successful team is one in which all members are held accountable. Accountability ensures that the team can not only meet their goals but also maintain avenues for open, transparent, and frequent communication and mutual respect amongst team members, but it can also be one of the challenging aspects of team management (Table 1) [17,18]. For each member to be accountable, expectations must be clear and reasonable. A regular meeting schedule can aid in maintaining accountability and provides the opportunity to give and receive feedback [11]. When setting up a meeting schedule, we suggest utilizing varied meeting formats (in person, virtual, hybrid), times, and days in order to determine what type of schedule fits the needs of the team. However, do not meet simply for the sake of meeting. Meetings should have a clear purpose with all members participating. Some examples of types of meeting formats are journal club meetings to learn new ideas and keep up to date in the field, quick status update meetings (status of papers, grants, administrative issues), and longer meetings to present works in progress to receive feedback among others [11,19,20]. Having an opportunity for the team to provide feedback is essential. Team leaders should be encouraged to provide feedback that is concrete, specific, descriptive, and fits into the shared goals of the team. In addition, in larger teams, developing a process to handle disagreements, before conflicts arise, may be beneficial. 

Formal, shared timelines are often helpful for presentations, abstracts, and publications to not only maintain accountability but ensure deliverables stay on track and are completed in a timely manner [21]. Lastly, take a cue from more defined trans-disciplinary teams such as SPORES [22], Comprehensive Cancer Centers [23], and the like that have both external and internal advisory boards. Advisory boards provide opportunities for new eyes to see the work and offer new perspectives and insights as well as the potential for new collaborations.

### 2.4. Credit

A key component of a successful team, in conjunction with accountability, is credit. How credit is handled can make or break a team (Table 1). Establish early in the team’s development the process that will be utilized to determine how authorship and how other forms of credit will be determined and shared. How will first and senior authorship be determined if it is not readily apparent [24,25]? Is there is a process/forum where members can raise concerns regarding credit? Who will present at local, national, and international conferences? In all presentations, the team members should be identified and specific contributions to the team acknowledged [24,25,26]. In addition, with large teams, it may be beneficial to determine who will be the “public contact”, the point person to whom any external questions around the team’s work will respond, which can often help in preventing mixed messages and conflicting priorities. 

### 2.5. Communication

Another stumbling block in the assembly of the team can be communication as the clinician and researcher, coming from different disciplines, may have their own unique vocabulary and definitions. These differences, which may not be readily apparent, have the potential to lead to misunderstandings and/or confusion amongst team members (Table 1). Both misunderstandings and confusion may occur at some point in the team and, when this does occur, team members should work together to resolve issues and learn from them. The resolution of miscommunication not only builds trust, but it also aids in the development of the team’s communication style. Therefore, differing opinions should not be avoided, as they may lead to new ideas. Team leaders should encourage the participation of all members and remind the team that disagreeing with an idea does not equate to a reprimand. A communication style that fosters active listening will develop trust. Further, the team should develop expectations of confidentiality so that all members feel comfortable sharing ideas and results knowing that these will not be communicated outside of the team without permission. As the team works together, the team communication style will develop and will ensure success, and innovation will become exponential. Lastly, while intra-team communication is an excellent starting point to build junior (trainees, graduate students, post-doctoral fellows, junior faculty members) skills and confidence, communication outside the group is also important [27]. Communication of the team’s ideas with others, not only through publications and presentations but also through team websites, can be utilized to promote the team internally and externally, including the general public. Grorud-Colvert and co-authors provide a strategy that, while focused on a different field, is applicable across the broader scientific community and includes (1) knowing your audience, (2) identifying the main message, (3) determining communication tactics, and (4) measuring success [28].

### 2.6. Leadership and Mentoring

As Vince Lombardi once said, “leaders are not born; they are made” [29]. Leadership is acquired and learned from much trial and error. An effective leader will bring together a group to develop ideas, coordinate efforts, and generate timelines for the team [30]. They will capitalize on the team’s strengths, providing an opportunity for all members to learn and exhibit their own leadership within the group [31]. As such, the team leaders should have the ability to recognize strengths as well as identify areas of growth. Mentoring and training team members help advance not only the individual but also the team. Building upon efficient communication, the leaders of the team should encourage feedback and discussion particularly from team members that are junior to them. By doing so, the leader will set a welcoming and encouraging tone to the group, increasing the sense of belonging. Effective leaders not only recognize the strengths of their team, but they also can identify areas where members have the potential to grow and encourage members to seek opportunities to do so. Mentoring can be formal, such as fostering undergraduate and graduate research [32], yet it does not need to be a formal active event. Passive mentoring occurs when members are allowed to observe how the leader prioritizes activities, develops ideas, communicates, and navigates various organizations. Strong team members build scientific, as well as personal, trust that can help propel both junior and senior scientists/clinicians. 

### 2.7. Proactively Dealing with Conflict

When working with more than one person, it is inevitable that conflict will arise at some point. How the team leaders react to this conflict is what will determine how well the team succeeds (Table 1). While conflict is never enjoyable, it should not be feared, as, when handled well and proactively, it can help move the team forward. Keep in mind the following when dealing with potential conflict: participate in active listening by being fully engaged in meetings and discussion, acknowledge others’ feelings, understand why members may be resistant to an idea, try to prevent internal competition by modeling cooperation, and solve problems in a collaborative manner [33,34]. 

One area of potential conflict may be ownership of data and/or resources [35]. With the continued and growing importance of resource sharing, developing data management and sharing (DMS) and resource sharing plans early on will not only fulfill NIH requirements [36] but will also address any potential issues/concerns for sharing prior to any publication, presentation, and especially for internal and external data requests. 

### 2.8. Fostering Forward Momentum

Science can often be unpredictable and slow. Revisiting and adjusting the team’s timelines and goals are critical to continue advancing the team and science. Check in with the team both informally and formally. By continually assessing shared goals, identifying and filling team gaps, and changing and evolving to meet the team’s needs, the team will maintain forward progress. All teams change over time; therefore, create a succession plan for changes in leadership and critical roles before they occur. Through its shared goals, accountability, and forward momentum, the team will be well poised to publish both early and often. These successes will aid in keeping both positivity and momentum. 

## 3. Conclusions

We hope this quick overview of key tactics (Figure 1), which support, interact, and build upon one another, will help the reader in their efforts in building or being part of a trans-disciplinary team. Trans-disciplinary teams can accelerate cancer research and improve the translation into effective treatments, practices, and policies. Team science not only impacts the immediate cancer-related science but also the science of generations to come as those junior investigators/clinicians go out to engage in their own team science. 

## Figures and Tables

**Table 1:** Common potential challenges in trans-disciplinary team science and suggested approaches/solutions.

Potential Challenges	Approaches/Solutions
Communication	Develop a shared team communication style; Be an active listener; Foster a “safe” team environment to share ideas, including expectations of confidentiality.
Dealing with Conflict	Be proactive—take action early on at the sign of conflict; Model cooperation; Solve problems collaboratively; Address conflict early; do not allow it to grow.
Credit	Agree on authorship early on in a project; Provide opportunities for sharing knowledge through manuscripts and presentations; Reassess authorship throughout projects; Clarify roles and responsibilities.
Accountability	Clearly lay out shared goals and expectations; Revisit goals/purpose of the team; Create project plans and timelines; Set a regular team meeting schedule; Ask for feedback and provide feedback (with kindness!).

## Data Availability

Not applicable.
